# Longitudinal developmental profiles in 4- to 5-year-old Dutch children: a latent profile analysis

**DOI:** 10.1007/s00787-026-03109-0

**Published:** 2026-07-08

**Authors:** Selena J. Rosinda, Pieter J. Hoekstra, Mijna Hadders-Algra, Kirsten R. Heineman, Annelies de Bildt

**Affiliations:** 1 Accare Child Study Center, Groningen, The Netherlands; 2 University of Groningen, University Medical Center Groningen, Department of Paediatrics, Beatrix Children’s Hospital, Division of Developmental Neurology, Groningen, The Netherlands; 3 University of Groningen, University Medical Center Groningen, Department of Psychiatry, Groningen, The Netherlands; 4 The Research School of Behavioural and Cognitive Neurosciences, University of Groningen, Groningen, The Netherlands; 5 University of Groningen, University Medical Center Groningen, Department of Paediatric Neurology, Groningen, The Netherlands; 6 Health in Context Research Institute, University Medical Center Groningen, University of Groningen, Groningen, The Netherlands

**Keywords:** Developmental profiles, Risk and protective factors, Typical development, Latent profile analysis

## Abstract

We aimed to gain knowledge on children’s development by distinguishing developmental profiles based on neurological and motor assessments in infancy, and motor and cognitive assessments at early school age. Profile characteristics and the associations between profiles and behavioural characteristics at early school age were also examined. Based on their neurological and motor scores at an age between 3 and 18 months, we invited 703 Dutch children of 4–5 years old (369 with atypical and 334 with typical infant neuromotor behaviour) of whom 518 (74%) participated in the current study. A latent profile analysis was performed to identify the developmental profiles. We examined profile characteristics and compared the parent-reported behavioural and emotional difficulties, ASD traits and ADHD traits at early school-age between the developmental profiles. We found four developmental profiles: ‘typical development’, ‘early advanced development’, ‘developmental catch-up’ and ‘developmental difficulties’. Being a boy was associated with the ‘developmental difficulties’ profile and being a girl and high parental educational level were associated with the ‘typical development’ profile. Children in the ‘developmental difficulties’ profile showed the most behavioural and emotional difficulties, ASD traits and ADHD traits The four profiles pointed to highly varying developmental patterns, underlining the inter- and intra-individual variability of development and showing the challenges of predicting later developmental outcome based on infancy assessments.

## Introduction

Children’s development is non-linear and continuously changing over time based on interactions between biological and environmental factors [1]. Hence, there is large heterogeneity between and within children. To capture this heterogeneity, it is helpful to establish longitudinal developmental profiles, which reflect subgroups of children with similar developmental patterns [2]. Performing such studies in population cohorts provides valuable information on both typical development and deviations from it, which allow to examine potential risk and protective factors for the different developmental patterns [2, 3]. A previous study on developmental language profiles indicated that while most preschoolers showed stable trajectories of typical language abilities over time, others showed fluctuating trajectories; their language abilities either accelerated or deteriorated (more than age appropriate) over time, underlining the intra-individual variability of development [4]. That study examined language development only. However, developmental domains are often interrelated. Thus focusing on, for example, both motor and cognitive domains, provides a better understanding of the child’s general development [5]. Other studies on developmental profiles, which did focus on multiple developmental domains simultaneously (either [a] cognitive and socio-emotional abilities; [b] physical, motor, socio-emotional/behavioural and communication/learning; or [c] motor, cognitive and verbal abilities), showed that children with developmental difficulties more frequently experienced attention deficit hyperactivity disorder (ADHD) symptoms and that their mothers more often had a lower educational level and socio-economic status than children in other profiles [6–8]. However, these studies focused on developmental domains measured either at one time point or longitudinally, always starting beyond infancy. Investigating multiple developmental domains that are representative of the child’s general development from infancy onwards is important as it may contribute to early detection and early intervention, which is most beneficial in infancy due to the high neuroplasticity during that period [9, 10].

Therefore, in the current study, we first aimed to distinguish developmental profiles by performing a latent profile analysis based on children’s neurological and motor development in infancy (as assessed by the Standardised Infant NeuroDevelopmental Assessment [SINDA] and the Infant Motor profile [IMP]), and their motor and cognitive development at 4 to 5 years of age (early school age; as assessed by the Movement Assessment Battery for Children [M-ABC] and the Wechsler Preschool and Primary Scale of Intelligence [WPPSI]). In infancy, the neurological and motor functioning of the child provide information on the child’s nervous system. These infant functions are important indicators for the child’s general development [11]. At early school age, cognitive and motor development are important indicators for general development, given that these domains include various capacities (e.g., working memory, verbal comprehension, visual-spatial and motor skills), which are useful in daily life [12]. Our second aim was to examine the profile characteristics based on child and environmental factors. Third, we examined whether there were differences in behavioural characteristics (behavioural and emotional difficulties, Autism Spectrum Disorder [ASD] traits and ADHD traits) at 4 to 5 years of age between the developmental profiles.

## Method

### Sample

The current sample was derived from a general population cohort of 1700 infants who were assessed with the Infant Motor Profile (IMP) [13] and the Standardized Infant NeuroDevelopmental Assessment (SINDA) [14] at an age between 2 and 18 months. More information on the original cohort has been published previously [13, 15]. From this cohort, we selected 703 children for the current study who were assessed in infancy between 3 and 18 months, as the IMP is applicable for this age [13]. We selected all 369 children with a potential risk for developmental problems, such as impairments in cognitive and neurological functioning and internalising problems [16], based on an atypical IMP (IMP total score below the 15th percentile and/or IMP variation and/or IMP performance scores below the 5th percentile) [13] and/or atypical SINDA score (i.e., 12 months or younger at assessment and a neurological scale score ≤ 21 and/or developmental scale score of ≤ 7) [14]. Furthermore, we selected 334 children with typical IMP (IMP total score above the 15th percentile and IMP variation and IMP performance scores above the 5th percentile) [13] and typical SINDA scores (13 to 18 months or 12 months or younger and a neurological scale score > 21 and developmental scale score > 7) [14]. For children with typical scores who did not want to participate, another child was invited to replace them, from the 897 children with typical IMP and SINDA scores, who were initially not selected for the follow-up study. For replacement, attention was paid to similarities in the assessment age in infancy and maternal educational level. The composition of the current population-based sample, with an oversampling of children with atypical infant neuromotor behaviour, allowed us to examine how infant neuromotor behaviour, based on the IMP and SINDA, is related to development at a later age.

At the follow-up, most parents (97%) completed the questionnaires, and most children had a completed WPPSI-IV-NL and M-ABC-2 (99%). For one child, only the parental questionnaires were completed but data on both the M-ABC-2 and the WPPSI-IV-NL were missing. Therefore, this child was omitted from the analyses. The current follow-up sample consisted of 517 children (74% of invited children) including 272 boys (53%) and 245 girls (47%). In infancy, the median assessment age was 10 months (range: 3–18) and at early school age it was 65 months (range: 58–71). All children completed the IMP and SINDA assessments in infancy. The children’s demographic characteristics and measurement scores are displayed in Table 1. Significantly more parents with a high educational level (mothers: 46% vs. 29%, χ2 = 28.6, *p* < .001; fathers: 42% vs. 25%, χ2 = 36.2, *p* < .001) and more fathers with a Dutch ethnic background (94% vs. 85%, χ2 = 17.8, *p* = .007) participated compared to the parents of invited children who did not participate. Additionally, there were significantly fewer children with atypical IMP or SINDA scores included in the current sample (43%) compared to the invited children who did not participate (80%, χ2 = 76.2, *p* < .001).

**Table 1:** Characteristics of the study sample (*n* = 517)

Assessment age in infancy in months; M (SD)	10 (5)
Assessment age at early school age in months; *M (SD)*	65 (3)
Sex: boy; *n* (%)	272 (53%)
Gestational age (weeks); median (range)	39.4 (29.1–42.1)
Small for gestational age^a^: yes; *n* (%)	54 (10%)
Birth weight (grams); *M* (*SD*)	3445 (597)
Non-optimal start at birth^b^: yes; *n* (%)	47 (9%)
Maternal educational level: low, middle, high^c^, *n* (%)	45 (9%), 226 (44%), 228 (44%)
Maternal ethnicity: Dutch^d^; *n* (%)	473 (91%)
Maternal illness/condition: yes^e^; *n* (%)	132 (25%)
Maternal smoking during pregnancy: yes^f^; *n* (%)	49 (10%)
Maternal hypertension during pregnancy: yes^f^; *n* (%)	53 (10%)
Gestational diabetes: yes^g^; *n* (%)	36 (7%)
Paternal educational level: low, middle, high^c^, *n* (%)	48 (9%), 233 (45%), 211 (41%)
Paternal ethnicity, Dutch^d^, *n* (%)	476 (92%)
Paternal illness/condition: yes^e^; *n* (%)	113 (22%)
IMP total score: median (range)	89 (61–99)
SINDA-23: median (range)	21 (9–23)
WPPSI-IV-NL total score; *M* (*SD*)	98 (14)
M-ABC-2 total score; *M* (*SD*)	69 (14)
SDQ – total difficulties score: median (range)	7 (0–37)
SRS-2 total score: median (range)	30 (3–136)
CPRS-R ADHD DSM-T^h^: median (range)	9 (0–52)

IMP = Infant Motor Profile, SINDA-23 = Standardized Infant NeuroDevelopmental Assessment - adapted neurological scale containing 23 items, WPPSI-IV-NL = Dutch Wechsler Preschool and Primary Scale of Intelligence – Fourth edition, M-ABC-2 = Movement Assessment Battery for Children – Second edition, SDQ = Strengths and Difficulties Questionnaire, SRS-2 = Social Responsiveness Scale – Second edition and CPRS-R = Conners Parent Rating Scale Revised.

^a^ low birth weight (< 10th percentile) for gestational age, ^b^ potential reasons: no immediate crying, respiratory complications, hypotonia or cyanosis, ^c^ low: no or primary school, pre-vocational secondary education, or secondary vocational education level 1, middle: senior general secondary education, pre-university education or vocational secondary education level 2–4, high: higher professional education and university education, ^d^ ‘*ethnical background…*’ based on self-report caregiver, ^e^ ‘*Do you have any known illness or condition?*’ based on self-report caregiver, ^f^ systolic > 140 mmHG or diastolic > 90 mmHG, based on self-report caregiver, ^g^ closed-ended question based on self-report caregiver, ^h^ Diagnostic and Statistical Manual total scale.

### Procedure

The selected children for our follow-up study were invited between July 2021 and March 2024, when they were 4 or 5 years old. The children participated in a two-and-a-half-hour assessment in our clinic or at home, based on the child’s and caregivers’ preferences. The video-recorded assessments included an intelligence test (Wechsler Preschool and Primary test of Intelligence; WPPSI-IV-NL) [12] and a motor test (Movement Assessment Battery for Children; M-ABC-2) [17]. The child’s caregivers completed an online questionnaire about their children’s behaviour in several domains (i.e., Social Responsiveness Scale 2 [18]; Strengths and Difficulties Questionnaire [19]; Conners Parent Rating Scale [20]) and gave written informed consent. The Medical Ethical Committee of the UMCG approved the study (METc 2020/546) and it was conducted in agreement with the Declaration of Helsinki, the General Data Protection Regulation (GDPR) and Dutch regulations and legislations regarding research.

### Measures

#### Assessments infancy

**Infant Motor Profile (IMP).** The IMP [13], a video-based clinical assessment for infants between 3 and 18 months, examines the quality of movements and motor milestones based on 80 items. The infant’s motor behaviour is evaluated on five domains: ‘Variation’ (25 items), ‘Adaptability’ (15 items), ‘Symmetry’ (10 items), ‘Fluency’ (7 items) and ‘Performance’ (i.e., motor skills, 23 items). We used only the age-dependent total score, i.e., the mean of the domain scores, except for children younger than six months for whom adaptability does not contribute to the total score. A score below the fifth percentile is considered at risk for neurodevelopmental disorders [13]. Previous studies showed high inter- and intrarater reliability of the IMP total score (ICC = 0.86 – 0.91 and 0.95, respectively) [21].

**Neurological Scale of the Standardised Infant NeuroDevelopmental Assessment (SINDA).** SINDA [14] is a neurodevelopmental assessment for infants aged 6 weeks up to and including 12 months. In this study, we only used SINDA’s neurological scale. Considering SINDA is validated until 12 months and the maximum infancy assessment age in the current sample was 18 months, we selected only the items from the scale’s 28 items that were applicable for the entire age range. This resulted in 23 items (SINDA-23), covering spontaneous movements (8 items), cranial nerves (7 items), muscle tone (4 items) and reflexes and reactions (4 items). Each item was scored as 0 = atypical or 1 = typical, and higher scores indicate a better neurological condition.

**Profile characteristics.** The following profile characteristics were examined, as they were found to be important for child development in previous research: the child’s sex [22], gestational age [23], being small for gestational age [23], birth weight [23], non-optimal start at birth [24], smoking during pregnancy [25], hypertension during pregnancy [26], gestational diabetes [27], parental educational level [28], parental ethnicity [29] and parental illness or condition [30]. Data on these factors were obtained by parental questionnaires during infancy and (if necessary, i.e., in case of complications during pregnancy or birth) from the infant’s medical records. The parental educational level and parental illness or condition was again asked during the follow-up assessments. Children who were small for gestational age had a lower birth weight (< 10th percentile) than expected for their gestational age. Potential reasons for a non-optimal start at birth could include no immediate crying, respiratory complications, hypotonia or cyanosis. The latter information was retrieved from medical records. Smoking during pregnancy, hypertension during pregnancy (systolic > 140 mmHG or diastolic > 90 mmHG) and gestational diabetes were asked through closed-ended questions (*‘no’* or *‘yes’*). Parental educational level was grouped into three groups: low (no or primary school, pre-vocational secondary education, or secondary vocational education level 1), middle (senior general secondary education, pre-university education or vocational secondary education level 2–4) and high (higher professional education and university education). Parental ethnicity was questioned with an open-ended statement: ‘*ethnical background mother/father …’*. Parental illness or condition was assessed with the question: *‘do you have any known illness or condition?’*, which could be answered with *‘no’* or *‘yes*,* namely…’*.

#### Assessments follow-up

**Movement Assessment Battery for Children 2 (M-ABC-2).** The M-ABC-2 is a clinical assessment of motor performance for children between 3 and 16 years [17, 31]. In approximately 30 min, we administered eight subtests to assess three motor components: ‘Manual dexterity’ (three subtests), ‘Aiming and catching’ (two subtests) and ‘Balance’ (three subtests). We used only the standardised total score. A score below the 5th percentile indicates a risk for developmental coordination disorder [31]. Previous studies showed high internal consistency (α = 0.83) and test-retest reliability (ICC = 0.83) [32].

**Wechsler Preschool and Primary Scale of Intelligence.** We used the Dutch version of the Wechsler Preschool and Primary Scale of Intelligence fourth edition (WPPSI-IV-NL) [12, 33], which is a clinical instrument that assesses cognitive abilities in children between 2:6 and 6:11 years. We administered the ten primary subtests that are available for children aged 4 years and older to determine five primary indices of intelligence (‘Verbal comprehension’, ‘Visual-spatial’, ‘Fluid reasoning’, ‘Working memory’ and ‘Processing speed’) and a total IQ score (TIQ) in approximately 60 min. We used TIQ > 85 as cut-off for developmental difficulties in the cognitive domain.

**Strengths and Difficulties Questionnaire (SDQ).** The parent-based SDQ, a screening questionnaire that addresses behavioural and emotional difficulties of children between 3 and 17 years old, contains 25 items divided over 5 subscales: (1) ‘Emotional symptoms’, (2) ‘Conduct problems’, (3) ‘Hyperactivity/inattention’, (4) ‘Peer relationship problems’ and (5) ‘Prosocial behaviour’ [19, 34]. We used the total difficulties score, based on the first four subscales. Each item was answered on a three-point Likert scale from 0 ‘Not true’ to 2 ‘Definitely true’. The cut-off score for behavioural and emotional difficulties is 12 or higher (four years old) and 15 or higher (five years old) [34]. Studies into the psychometric properties of the SDQ showed high internal consistency (α = .77 – .82) and test-retest reliability (*r* = .77) for the total score [35].

**Social Responsiveness Scale-2 (SRS-2).** The SRS-2 is a parental screening questionnaire for autism spectrum disorder (ASD) in children from 4 to 18 years, containing 65 items with a four-point Likert scale ranging from 0 ‘Not true’ to 3 ‘Almost always true’ [18]. Children in the general population with a score of 70 or higher have an increased risk of ASD [18]. The SRS-2 is reliable (α = 0.94 – 0.96, *r =* .77) and has good predictive values for ASD (sensitivity and specificity = 0.92) [36].

**Conners Parent Rating Scale Revised long version (CPRS-R).** The long version of the parent-reported CPRS-R consists of 80 items that assess behavioural problems (e.g., ADHD related behaviour) of children between 3 and 17 years old [20]. We used the Diagnostic and Statistical Manual of Mental Disorders (DSM) total ADHD subscale from the CPRS-R, which consists of 18 items on hyperactivity and inattention and are answered on a four-point Likert scale ranging from 0 ‘Not at all true’ to 3 ‘Very much true’. Children with a t-score of 65 or higher have an increased risk of ADHD [37]. The CPRS-R is internally consistent (α = 0.77 – 0.92) and has good predictive values for ADHD (sensitivity = 0.92, specificity = 0.94) [20].

### Statistical analyses

First, we compared the characteristics of our sample and the invited children who did not participate in our follow-up study by using independent sample t-tests and chi-square tests. Then, to distinguish developmental profiles, a latent profile analysis was performed in Mplus (version 8.11, DEMO) [38]. The indicator variables were standardised scores (Z-scores) of motor and neurological assessments in infancy (IMP total and SINDA-23, respectively) and motor and cognitive assessments at follow-up (M-ABC-2 total and WPPSI-IV-NL total scales, respectively). Subsequently, we compared two to eight profile solutions with varying means. We explored if variances should be equal and covariances should be fixed to zero or allowed to vary. To avoid local maxima, we increased the random sets of starting values (initial stage to 100, final stage optimisations to 20 and initial stage iterations to 50) and ended comparing additional profiles if the loglikelihood value was not replicated or if the model was unidentified. To determine the optimal solution, we compared model fit statistics (Bayesian information criterion [BIC] and Akaike information criterion [AIC]) between the models with different parameter constraints. Additionally, classification accuracy (entropy and classification proportion), a bootstrapped likelihood ratio test and profile plots (interpretation of each profile) were compared within models with the same parameter constraints but with a different number of profiles. A more optimal solution was characterised by a trade-off between lower BIC and AIC values, higher entropy, profile size of at least 1% of the total sample and a significant bootstrapped likelihood ratio test [39]. Then, we performed Fisher (-Freeman-Halton) exact tests (including post-hoc tests based on the adjusted residuals) and an Analysis of Variance (ANOVA) test (including post-hoc test) to investigate characteristics of the developmental profiles based on child factors (assessment age infancy, assessment age follow-up, sex, gestational age, small for gestational age and non-optimal start at birth) and environmental factors (parental ethnicity, parental educational level, parental illness or condition, smoking during pregnancy, gestational diabetes and maternal hypertension during pregnancy). Additionally, we examined if the profiles differed regarding behavioural characteristics at early school age (behavioural and emotional difficulties measured with the SDQ total difficulties scale, ASD traits measured with the SRS-2 total scale and ADHD traits measured with the CPRS-R DSM total ADHD scale) through Kruskal-Wallis tests with post-hoc tests. An alpha level of 0.05 was considered statistically significant. All analyses, except for the latent profile analysis, were completed in IBM SPSS statistics (version 28.0.1.0).

## Results

### Model selection and optimal solution

Table 2 presents the fit statistics of all explored models. We first examined the BIC values of the models. The model with the lowest BIC value (2-profiles solution in model without constraints) did not adequately fit the data, as the bootstrap likelihood test showed that a 3-profiles solution in the model without constraints was a better fit. However, for that model the BIC was higher, indicating a poorer model fit. Therefore, we examined the model with the second lowest BIC. This was the 4-profiles solution in the model with varying means, varying variances and covariances fixed to zero as the best model. This model also had a relatively low AIC, a significant bootstrapped likelihood ratio test and accurate sample sizes in each profile. Additionally, the developmental profiles seemed theoretically plausible. Therefore, we chose the 4-profiles in the model with varying mean, varying variances and covariances fixed to zero as the optimal solution.

**Table 2:** Model fit statistics for k-profile solutions

*Models with varying means, equal variances and covariances zero*					
	LL	AIC	BIC	Entropy	*p*_blrt_
2-profiles	-2811	5648	5703	0.816	< 0.001
3-profiles	-2775	5587	5663	0.860	< 0.001
4-profiles	Unidentified model				
*Models with varying means, varying variances and covariances zero*					
	LL	AIC	BIC	Entropy	*p*_blrt_
2-profiles	-2770	5573	5645	0.671	< 0.001
3-profiles	-2728	5509	5619	0.608	< 0.001
4-profiles	-2690	5451	5599	0.677	< 0.001
5-profiles	Not replicated				
*Models with varying means, varying variances and varying covariances*					
	LL	AIC	BIC	Entropy	*p*_blrt_
2-profiles	-2705	5469	5592	0.558	< 0.001
3-profiles	-2673	5435	5622	0.672	< 0.001
4-profiles	-2648	5415	5666	0.684	0.600
5-profiles	Not replicated				

LL = Loglikelihood value, AIC = Akaike Information Criterion, BIC = Bayesian Information Criterion, *p*_blrt_ = *p*-value of the bootstrapped likelihood ratio test

**Fig. 1:**
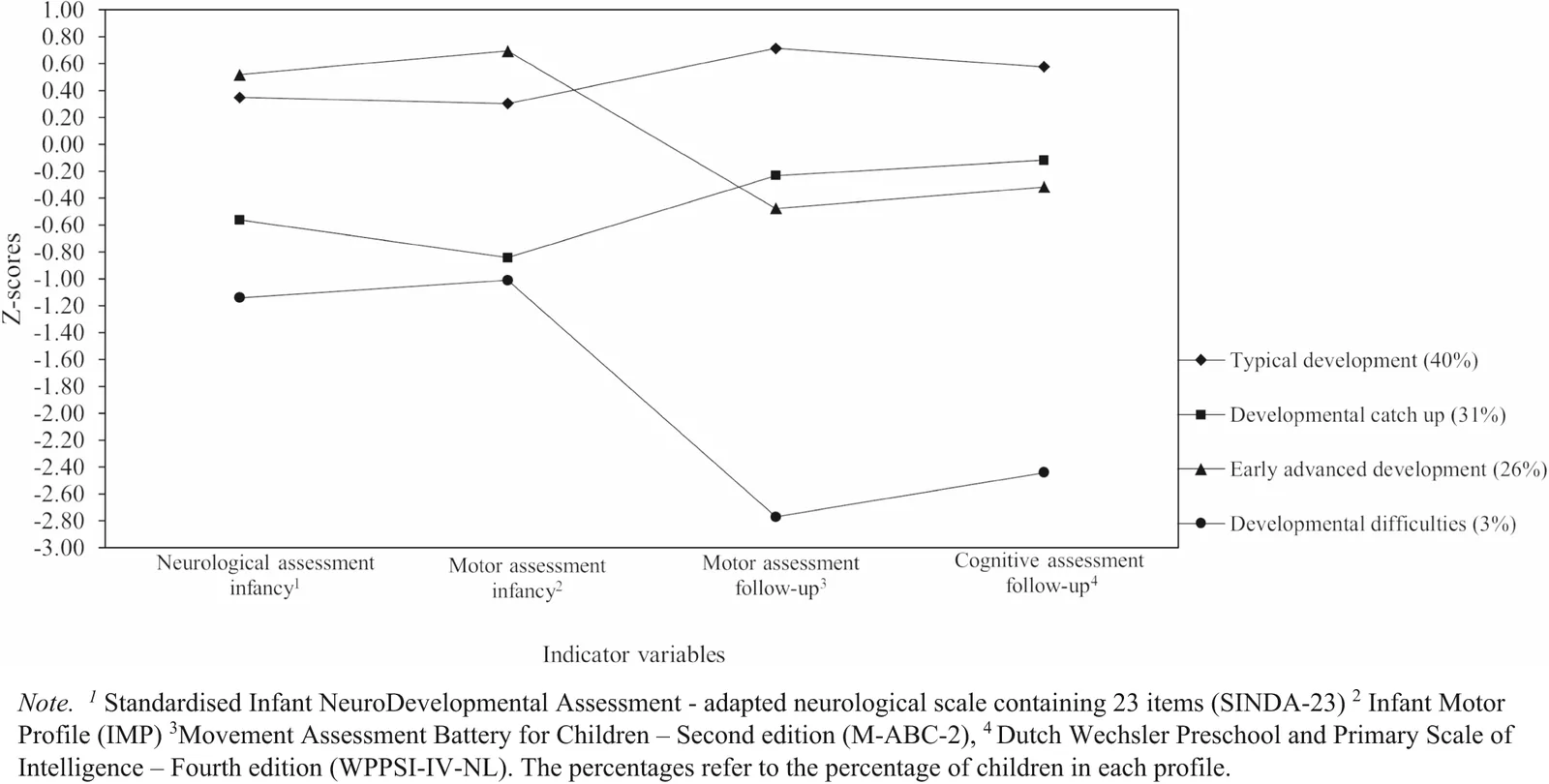
Profile plot optimal solution: representing four developmental profiles

Figure 1 presents the profile plot of the optimal solution. Information regarding the scores of the indicator variables in each profile is displayed in Table 3. Children in the first profile (40% of the total sample), named ‘typical development’, scored average in infancy and above average at early school age. In the second profile, the ‘developmental catch-up’ profile, containing 31% of the total sample, children had below average scores in infancy and average scores at early school age. Children in the third profile (26% of the total sample), the ‘early advanced development’ profile, scored above average on the infancy assessments, yet they scored lower (although average) on the school-age assessments. Finally, children in the fourth profile (3% of the total sample), named ‘developmental difficulties’, showed low scores on the infancy assessments and even lower scores at early school age.

**Table 3:** Scores indicator variables in each developmental profile

	Typical development (*n* = 207)	Developmental catch-up (*n* = 163)	Early advanced development (*n* = 134)	Developmental difficulties (*n* = 13)
Neurological assessment infancy^1^				
*Z* (*S*^2^)	0.349 (0.375)	− 0.563 (1.07)	0.519 (0.261)	-1.14 (3.39)
*M* (*SD*)	21.4 (1.21)	19.5 (2.03)	21.8 (0.992)	18.6 (3.82)
% below cut-off	*	*	*	*
Motor assessment infancy^2^				
*Z* (*S*^2^)	0.305 (0.648)	− 0.843 (0.535)	0.695 (0.354)	-1.01 (1.11)
*M* (*SD*)	**	**	**	**
% below cut-off	2%	15%	0%	38%
Motor assessment follow-up^3^				
*Z* (*S*^2^)	0.715 (0.269)	− 0.233 (0.649)	− 0.477 (0.796)	-2.77 (0.586)
*M* (*SD*)	79.3 (6.56)	64.5 (10.8)	59.9 (11.0)	29.3 (10.3)
% below cut-off	0%	15%	25%	100%
Cognitive assessment follow-up^4^				
*Z* (*S*^2^)	0.577 (0.711)	− 0.118 (0.766)	− 0.316 (0.608)	-2.44 (0.780)
*M* (*SD*)	107 (11.0)	95.9 (12.0)	91.8 (10.5)	63.5 (12.4)
% below cut-off	2%	18%	28%	100%

^*1*^ Standardised Infant Neuro Developmental Assessment - adapted neurological scale containing 23 items (SINDA-23) ^2^ Infant Motor Profile (IMP) ^3^Movement Assessment Battery for Children – Second edition (M-ABC-2) ^4^ Dutch Wechsler Preschool and Primary Scale of Intelligence – Fourth edition (WPPSI-IV-NL)

*Z = mean z-score*, S^2^* = variance, M = mean, SD = standard deviation*

Cut-offs are indicative for developmental difficulties on the concerning developmental domain (motor and cognitive domains, respectively): IMP total score = below 5th percentile, M-ABC-2 total = below 5th percentile and WPPSI-IV-NL total index score = below 85

* Unavailable for SINDA-23 ** not displayed as this score is dependent on the assessment age, which varied between children in different profiles

### Characteristics of the developmental profiles

The child’s sex, gestational age, being small for gestational age, maternal educational level and paternal educational level differed significantly between the profiles (*p* = .002, *p* = .010, *p* = .008, *p* = .003 and *p* < .001, respectively; see Table 4). The ‘early advanced development’ profile contained significantly more boys (62%) compared to the ‘typical development’ and ‘developmental catch-up’ (43% and 55%, respectively) profiles, but significantly less boys than the ’developmental difficulties’ profile (77%). The ‘typical development’ profile contained significantly more girls (57%) compared to all other profiles (23% – 45%). Children in the ‘early advanced development’ and ‘typical development’ profiles had a significantly higher gestational age than children in the ‘developmental catch-up’ profile (*F*(3, 516) = 3.847, *p* = .010). Significantly fewer children were small for gestational age in the ‘typical development’ profile than in the other profiles (5% vs. 13% – 15%) and the ‘early advanced development’ profile contained more children who were small for gestational age (15%) than children in the ‘developmental catch-up’ profile (13%).

Furthermore, in the ‘typical development’ profile, children more often had mothers (56% vs. 35% – 44%) and fathers (57% vs. 25% – 36%) with a high educational level than children in the other profiles. Children in the ‘developmental catch-up’ profile had more often a father with a high educational level (36%) than children in the ‘early advanced development’ and ‘developmental difficulties’ profiles (31% and 25%, respectively) and less often a father with a middle educational level (51%) compared to children in the ‘early advanced development’ profile (56%). Lastly, children in the ‘early advanced development’ profile less often had a mother with a high educational level (35% vs 39% – 56%) and more often a mother with a middle educational level (54% vs 39% – 48%) than children in the other profiles. The other characteristics did not differ significantly between the developmental profiles.

**Table 4:** Significant differences in profile characteristics and in behavioural and emotional difficulties, ADHD traits and ASD traits at early school age between the developmental profiles

	Typical development^1^ (*n* = 207)	Developmental catch-up^2^ (*n* = 163)	Early advanced development^3^ (*n* = 134)	Developmental difficulties^4^ (*n* = 13)	Significant group differences (*p*-value)
Gestational age					
M (SD)	39.6 (1.66)	39.0 (1.96)	39.5 (1.83)	39.3 (1.33)	1 > 2 (*p*=.001); 3 > 2 (*p=*.015)
Small for gestational age					
Yes No	11 (5%) 196 (95%)	21 (13%) 142 (87%)	20 (15%) 113 (84%)	2 (15%) 11 (85%)	2,3,4 > 1 (*p=*.002); 3 > 2 (*p*=.046)
Child’s sex					
Boys Girls	90 (43%) 117 (57%)	89 (55%) 74 (45%)	83 (62%) 51 (38%)	10 (77%) 3 (23%)	3 > 1,2 (*p*=.012); 4 > 3 (*p*=.012) 1 > 2,3,4 (*p*=.001)
Maternal education level^a^					
Low Middle High	10 (5%) 79 (38%) 111 (54%)	17 (10%) 72 (44%) 69 (42%)	16 (12%) 69 (51%) 44 (33%)	2 (15%) 6 (46%) 4 (31%)	2,3,4 > 1 (*p=.*010) 2,3,4 > 1 (*p=.*034); 3 > 1,2,4 (*p=*.030) 1 > 2,3,4 (*p*<.001); 1,2 > 3 (*p=*.002)
Paternal education level^a^					
Low Middle High	10 (5%) 75 (36%) 112 (54%)	20 (12%) 79 (48%) 56 (34%)	16 (12%) 72 (54%) 40 (30%)	2 (15%) 7 (54%) 3 (23%)	2,3,4 > 1 (*p*=.004) 2,3,4 > 1 (*p*=.001); 3 > 2 (*p* = .019) 1 > 2,3,4 (*p*<.001); 2 > 3,4(*p*=.040); 3 > 4 (*p*=.002)
SDQ total difficulties					
*M* (*SD*) % above cut-off	6.93 (5.25) 10%	7.91 (4.65) 11%	9.84 (6.18) 15%	11.6 (5.66) 31%	2,3,4 > 1(*p*<.001–0.021); 3 > 2 (*p*=.015); 4 > 2(*p*=.029)
SRS-2 total					
*M* (*SD*) % above cut-off	29.5 (17.1) 2%	35.4 (18.0) 5%	38.6 (23.5) 8%	64.9 (38.6) 31%	2,3,4 > 1(*p*<.001–0.002); 4 > 2,3 (*p*=.003–0.008)
CPRS-R ADHD DSM-T					
*M* (*SD*) % above cut-off	9.62 (8.19) 8%	11.82 (7.59) 9%	12.9 (10.2) 14%	20.7 (12.1) 39%	2,3,4 < 1 (*p*<.001–0.001); 4 > 2,3 (*p*=.013–0.015)

^a^ low: no or primary school, pre-vocational secondary education, or secondary vocational education level 1, middle: senior general secondary education, pre-university education or vocational secondary education level 2–4, high: higher professional education and university education

SDQ = Strengths and Difficulties Questionnaire, SRS-2 = Social Responsiveness Scale – Second edition and CPRS-R = Conners Parent Rating Scale Revised, *M* = mean, *SD =* standard deviation

Cut-off behavioural and emotional difficulties: 12 or higher for four-year-olds and 15 or higher for five-year-olds, cut-off ASD traits: 70 or higher, cut-off ADHD traits: t-score of 65 or higher

### Developmental profiles and behavioural characteristics

The parent-reported behavioural and emotional difficulties, ASD traits and ADHD traits at early school age differed significantly between the profiles (χ^2^(3) = 27.891, *p* < .001; χ^2^(3) = 32.514, *p* < .001; χ^2^(3) = 25.041, *p* < .001, respectively; Table 4). The post-hoc tests showed that children in the ‘early advanced development’, ‘developmental catch-up’ and ‘developmental difficulties’ profiles showed significantly more behavioural and emotional difficulties, ASD traits and ADHD traits than children in the ‘typical development’ profile. Furthermore, children in the ‘early advanced development’ and ‘developmental difficulties’ profiles displayed significantly more behavioural and emotional difficulties than children in the ‘developmental catch-up’ profile. Lastly, children in the ‘developmental difficulties’ profile showed significantly more ASD traits and ADHD traits than children in the ‘early advanced development’ and ‘developmental catch-up’ profiles.

## Discussion

Based on neurological and motor assessments in infancy and motor and cognitive assessments at early school age, we identified four developmental profiles in a sample of 4- to 5-year-old children from the general population, with an oversampling of children with atypical infant neuromotor behaviour. When interpreting the results, it is important to take into account that the proportion of children in each profile is only applicable to our study and not necessarily to the general population. Particularly, we found that, in our sample, most children developed age-appropriately both in infancy and at early school age (‘typical development’ profile). Other children showed a developmental advantage in infancy, while at early school age they scored similarly as their peers (‘early advanced development’ profile). Additionally, we found that one group of children who developed below average in infancy showed a typical development at early school age (‘developmental catch-up’ profile). Only few children showed developmental difficulties at both time points (‘developmental difficulties’ profile). The latter developmental pattern may be compared to the phenomenon ‘growing into deficit’, in which developmental or neurological difficulties become more evident over time [40].

The varying developmental patterns that were found in the current study underline the uncertainty of predicting later development based solely on infancy assessments and stress the importance of monitoring development over time [9]. The difficulties to predict the child’s developmental course from infancy assessments may be explained by the large developmental changes in the brain during early infancy, such as the substitution of temporary neural structures (e.g., the cortical subplate) for permanent cortical structures, which is only completed by the end of the first year [41]. Prediction of developmental outcome may therefore be better after the first year [42].

Furthermore, within our sample, being a boy was associated with the ‘developmental difficulties’ profile, which is in line with previous research on risk factors for neurodevelopmental conditions [43]. High parental educational levels and being a girl were associated with typical development, which is also consistent with the literature [43, 44]. Unexpectedly, gestational age was significantly higher in children in the ‘early advanced development’ profile than in the ‘developmental catch-up’ profile. Children in the ‘early advanced development’ profile had a more optimal development in infancy (illustrated by higher average IMP and SINDA scores) compared to children in the ‘developmental catch-up’ profile. A previous study indicated that a higher gestational age at term was associated with higher IMP total and SINDA developmental scores [45]. Possibly, a higher gestational age at term results in a temporary developmental advantage during infancy, which might not persist into later age. Notably, there were no significant characteristics for children in the ‘early advanced development’ profile that may explain why the developmental scores of these children were lower at early school age than in infancy. Perhaps other, unknown environmental factors might explain why the children with a developmental advantage in infancy scored on average at early school age [46].

Finally, in our sample, behavioural and emotional difficulties, ASD traits and ADHD traits were not limited to children in whom neurological, motor and/or cognitive difficulties were evident. Children in the ‘typical development’ ‘early advanced development’ and ‘developmental catch-up’ profiles also showed behavioural and emotional difficulties, ASD traits and ADHD traits, despite the lack of neurological, motor and/or cognitive difficulties at one or both time points. This underlines the importance of incorporating a dimensional approach in developmental research, examining characteristics of children’s development on a continuum instead of in a categorical manner [47]. Nonetheless, children with neurological, motor and/or cognitive difficulties showed the most behavioural and emotional difficulties, ASD traits and ADHD traits. Especially the combination of these difficulties may impact the child’s wellbeing, which emphasizes the importance of early detection and intervention [48].

### Strengths, limitations and future research

A strength of the current study is that the child’s development was assessed in infancy and at early school age, with a reasonable sample size. However, a limitation is the generalisability of the results due to the composition of the current sample. From a population-based sample, we selected all children with atypical IMP and SINDA scores and only 27% of children with typical IMP and SINDA scores to participate in the follow-up study. Therefore, the current sample was no longer representative of the general population. Furthermore, for the interpretation of the results it is important to consider that the ‘developmental difficulties’ profile only contained 13 children. Nonetheless, the profile contains more than 1% of children in our sample [39] and seems theoretically plausible, as only few children will be expected to have developmental difficulties as our sample was derived from a population-based cohort. Moreover, the entropy value of the optimal solution is .677. Although the current entropy value exceeds the suggested minimum of .600 [49], it indicates the accuracy of assigning a child to a certain profile is not fully refined. Additionally, the assessments’ duration at early school age (2.5 hours) may have impacted the child’s performance due to tiredness or diminished concentration [50]. Lastly, in the current study we used SINDA-23 (only including the neurological SINDA items that are age-independent between 3 and 18 months) which has not been validated. Future studies on developmental profiles should examine the developmental course of children from a general population sample with more frequent time points and consider environmental factors that may affect the child’s development.

## Conclusion

We identified four distinct developmental profiles in our sample based on neurological and motor assessments in infancy and motor and cognitive assessments at early school age. While some children developed typically or had developmental difficulties at both time points, other children showed a more inconsistent development when comparing the assessment scores at the two time points. The four profiles underline the inter- and intra-individual variability of development. Additionally, the divergent developmental patterns in the profiles show the challenges involved in the prediction of later developmental outcomes, especially when the first assessment occurs at an early age. Hence, it is important to monitor children’s development. Consistent with previous studies, we found that being a boy was associated with developmental difficulties, while being a girl and high parental educational level were associated with typical development. Altogether, this study enhances our understanding of typical development patterns, its variations from infancy onwards, and their associated profile characteristics and behaviours. Future studies are necessary to replicate the current findings in samples representative of the general population.

## Data Availability

The datasets generated and analysed during the current study are not publicly available due to privacy and ethical restrictions but are available from the corresponding author on reasonable request.

## References

[1] Thelen E (2005) Dynamic systems theory and the complexity of change. Psychoanal Dialogues 15:255–283. 10.1080/10481881509348831

[2] van der Gaag MAE (2023) A person-centered approach in developmental science: Why this is the future and how to get there. Infant Child Dev 32:e2478. 10.1002/ICD.2478

[3] Volkow ND, Gordon JA, Bianchi DW et al (2024) The healthy brain and child development study (HBCD): NIH collaboration to understand the impacts of prenatal and early life experiences on brain development. Dev Cogn Neurosci 69:101423. 10.1016/J.DCN.2024.101423 PMC11342761 39098249

[4] Ukoumunne OC, Wake M, Carlin J et al (2012) Profiles of language development in pre-school children: A longitudinal latent class analysis of data from the Early Language in Victoria Study. Child Care Health Dev 38:341–349. 10.1111/J.1365-2214.2011.01234.X 21434972

[5] D’souza D, D’souza H, Karmiloff-Smith A (2017) Precursors to language development in typically and atypically developing infants and toddlers: the importance of embracing complexity. J Child Lang 44:591–627. 10.1017/S030500091700006X 28393740

[6] Black M, Adjei NK, Strong M et al (2023) Trajectories of child cognitive and socioemotional development and associations with adolescent health in the uk millennium cohort study. J Pediatr 263:113611. 10.1016/j.jpeds.2023.113611 37468036

[7] Russell J, Grant CC, Morton S et al (2023) Prevalence and predictors of developmental health difficulties within New Zealand preschool-aged children: a latent profile analysis. J R Soc N Z 53:587–614. 10.1080/03036758.2022.2083188 PMC11459816 39440132

[8] Houwen S, Kamphorst E, van der Veer G, Cantell M (2019) Identifying patterns of motor performance, executive functioning, and verbal ability in preschool children: A latent profile analysis. Res Dev Disabil 84:3–15. 10.1016/J.RIDD.2018.04.002 29724641

[9] Lipkin PH, Macias MM, Hyman SL et al (2020) Promoting optimal development: Identifying infants and young children with developmental disorders through developmental surveillance and screening. Pediatrics 145:e20193449. 10.1542/PEDS.2019-3449/36971 31843861

[10] Hadders-Algra M (2021) Early detection and early intervention in developmental motor disorders. From Neuroscience to Participation. Mac Keith

[11] Hadders-Algra M (2002) Variability in infant motor behavior: A hallmark of the healthy nervous system. Infant Behav Dev 25:433–451. 10.1016/S0163-6383(02)00144-3

[12] Wechsler D, Hurks P, Hendriksen J (2020) Wechsler Preschool and Primary Scale of Intelligence - Fourth edition - Nederlandstalige bewerking: Technische handleiding. Pearson Benelux, Amsterdam

[13] Hadders-Algra M, Heineman KR (2021) The Infant Motor Profile. Routeledge, London

[14] Hadders-Algra M, Tacke U, Pietz J, Philippi H (2022) SINDA: Standardized infant neurodevelopmental assessment: an instrument for early detection of neurodevelopmental disorders. Mac Keith, London

[15] Straathof EJM, Heineman KR, Hamer EG, Hadders-Algra M (2020) Prevailing head position to one side in early infancy—A population-based study. Acta Paediatr 109:1423–1429. 10.1111/apa.15112 PMC7318227 31782830

[16] Wu YC, Heineman KR, La Bastide-Van Gemert S et al (2020) Motor behaviour in infancy is associated with neurological, cognitive, and behavioural function of children born to parents with reduced fertility. Dev Med Child Neurol 62:1089–1095. 10.1111/DMCN.14520 PMC7496844 32222973

[17] Smits-Engelsman B (2010) Movement ABC-2 NL | Movement assessment battery for children, 2nd edn. Pearson Benelux, Amsterdam

[18] Constantino JN, Gruber CP (2012) Social responsiveness scale, Second Edition (SRS-2). Western Psychological Services, Torrance

[19] Goodman R (1997) The strengths and difficulties questionnaire: a research note. J Child Psychol Psychiatry 38:581–586. 10.1111/j.1469-7610.1997.tb01545.x 9255702

[20] Conners KC, Sitarenios G, Parker JDA, Epstein JN (1998) The revised conners’ parent rating scale (CPRS-R): factor structure, reliability, and criterion validity. J Abnorm Child Psychol 26:257–268. 10.1023/A:1022602400621 9700518

[21] Tveten KM, Hadders-Algra M, Strand LI et al (2020) Intra- and inter-rater reliability of the infant motor profile in infants in primary health care. Phys Occup Ther Pediatr 40:571–581. 10.1080/01942638.2020.1720331 32003280

[22] Giofrè D, Allen K, Toffalini E, Caviola S (2022) The impasse on gender differences in intelligence: a meta-analysis on WISC batteries. Edu Psychol Rev 34:2543–2568. 10.1007/s10648-022-09705-1

[23] Gonçalves R, Blauuwendraad S, Avraam D et al (2025) Early-life growth and emotional, behavior and cognitive outcomes in childhood and adolescence in the EU child cohort network: individual participant data meta-analysis of over 109,00 individuals. Lancet Reg Health Eur 52:101247. 10.1016/j.lanepe.2025.101247 PMC11910110 40094119

[24] Dzikienė R, Lukoševičius S, Laurynaitienė J, Marmienė V, Nedzelskienė I, Tamelienė R, Rimdeikienė I, Kudrevičienė A (2021) Long-term outcomes of perinatal hypoxia and asphyxia at an early school age. Medicina 57:988. 10.3390/medicina57090988 PMC8466311 34577911

[25] Godleski S, Shisler S, Colton K, Leising M (2024) Prenatal tobacco exposure and behavioral disorders in children and adolescents: Systematic review and meta-analysis. Pediatr Rep 16:736–752. 10.3390/pediatric16030062 PMC11417955 39311325

[26] Atkinson J, Gordon H, Tong S, Walker S, Mehdipour P, Lindquist A, Hastle R (2025) Hypertensive disorders of pregnancy and childhood neurodevelopment: A systematic review and meta-analysis. Plos Med 22:e1004558. 10.1371/journal.pmed.1004558 PMC12422451 40929171

[27] Pretorius R, Avraam D, Guxens M et al (2025) Is maternal diabetes during pregnancy associated with neurodevelopmental, cognitive and behavioural outcomes in children? Inisghts from individual participant data meta-analysis in ten birth cohorts. BMC Pediatr 25:76. 10.1186/s12887-024-05365-y PMC11783732 39885386

[28] Tamayo Martinez N, Xerxa Y, Law J, Serdarevic F, Jansen PW, Tiemeier H, Double (2022) Advantage of parental education for child educational achievement: the role of parenting and child intelligence. Eur J Public Health 32:690–695. 10.1093/eurpub/ckac044 PMC9527951 35554528

[29] Lansford J (2025) Theories of parenting and child development in different cultural contexts. Theory Soc 54:851–865. 10.1007/s11186-025-09639-x PMC12916138 41716302

[30] Stracke M, Heinzl M, Müller A, Gilbert K, Thorup A, Paul J, Christiansen H (2023) Mental health is a family affair – Systematic review and meta-analysis on the associations between mental health problems in parents and children during the COVID-19 pandemic. Int J Environ Res Public Health 20:4485. 10.3390/ijerph20054485 PMC10001622 36901492

[31] Henderson SE, Sugden D, Barnett AL (2007) Movement assessment battery for children-2, 2nd edn. Harcourt Assessment, London

[32] Ghayour Najafabadi M, Saghaei B, Shariat A et al (2022) Validity and reliability of the movement assessment battery second edition test in children with and without motor impairment: A prospective cohort study. Ann Med Surg 77:103672. 10.1016/J.AMSU.2022.103672 PMC9142614 35638021

[33] Wechsler D (2012) Wechsler Preschool and Primary Scale of Intelligence - Fourth Edition (WPPSI-IV): Administration and scoring manual, Fourth. The Psychological Cooperation, San Antonio

[34] Theunissen MHC, De Wolff MS, Van Grieken A, Mieloo C (2016) Handleiding voor het gebruik van de Strengths and Difficulties Questionnaire binnen de Jeugdgezondheidszorg. TNO, Leiden

[35] Stone LL, Otten R, Engels RCME et al (2010) Psychometric properties of the parent and teacher versions of the strengths and difficulties questionnaire for 4- to 12-Year-olds: A review. Clin Child Fam Psychol Rev 13:254–274. 10.1007/S10567-010-0071-2 PMC2919684 20589428

[36] Bruni TP (2014) Test review: social responsiveness scale–second edition (SRS-2). J Psychoeduc Assess 32:365–369. 10.1177/0734282913517525

[37] Collett BR, Ohan JL, Myers KM (2003) Ten-year review of rating scales. V: Scales assessing attention-deficit/ hyperactivity disorder. J Am Acad Child Adolesc Psychiatry 42:1015–1037. 10.1097/01.CHI.0000070245.24125.B6 12960702

[38] Muthén LK, Muthén BO (2017) Mplus

[39] Spurk D, Hirschi A, Wang M et al (2020) Latent profile analysis: A review and how to guide of its application within vocational behavior research. J Vocat Behav 120:103445. 10.1016/j.jvb.2020.103445

[40] Vles (2000) Growing into deficit. Maastricht University, Maastricht

[41] Hadders-Algra M (2021) Early diagnostics and early intervention in neurodevelopmental disorders—age-dependent challenges and opportunities. J Clin Med 10:861–883. 10.3390/jcm10040861 PMC7922888 33669727

[42] De Graaf-Peters VB, Hadders-Algra M (2006) Ontogeny of the human central nervous system: What is happening when? Early Hum Dev 82:257–266. 10.1016/j.earlhumdev.2005.10.013 16360292

[43] Bölte S, Neufeld J, Marschik PB et al (2023) Sex and gender in neurodevelopmental conditions. Nat Rev Neurol 19:136–159. 10.1038/s41582-023-00774-6 PMC10154737 36747038

[44] Putnick DL, Perkins NJ, Yeung E, Peddada SD (2024) Do maternal and paternal education protect against developmental delays? Res Dev Disabil 154:104848. 10.1016/J.RIDD.2024.104848 PMC11560501 39332281

[45] Hadders-Algra M, Van Iersel PAM, Heineman KR, La Bastide-Van Gemert S (2023) Longer duration of gestation in term singletons is associated with better infant neurodevelopment. J Earl Hum Dev 181:105779. 10.1016/j.earlyhumdev.2023.105779 37120903

[46] Bronfenbrenner U (1979) The ecology of human development: experiments by nature and design. Harvard University Press, Cambridge, MA

[47] Astle DE, Holmes J, Kievit R, Gathercole SE (2022) Annual research review: the transdiagnostic revolution in neurodevelopmental disorders. J Child Psychol Psychiatry 63:397–417. 10.1111/JCPP.13481 34296774

[48] Roberts C, Mazzucchelli T, Taylor K, Reid R (2003) Early intervention for behaviour problems in young children with developmental disabilities. Int J Disabil Dev Educ 50:275–292. 10.1080/1034912032000120453

[49] Bauer J (2022) A primer to latent profile and latent class analysis. In: Goller M, Kyndt E, Paloniemi S, Damsa A (eds) Methods for researching professional learning and development. Springer, Cham, pp 243–268

[50] Greher MR, Wodushek TR (2017) Performance validity testing in neuropsychology: scientific basis and clinical application-a brief review. J Psychiatr Pract 23:134–140. 10.1097/PRA.0000000000000218 28291039

